# Release of silicone oil droplets from syringes

**DOI:** 10.1186/s40942-018-0153-8

**Published:** 2019-01-03

**Authors:** Gustavo Barreto Melo, Celso de Souza Dias Junior, Mariana Reis Carvalho, Alexandre Lima Cardoso, Fábio Barreto Morais, Ana Carolina Migliorini Figueira, Acácio Alves Souza Lima Filho, Geoffrey Guy Emerson, Maurício Maia

**Affiliations:** 1 Hospital de Olhos de Sergipe, Rua Campo do Brito, 995 São José, 49020-380 Aracaju, SE Brazil; 2 Federal University of São Paulo, São Paulo, SP Brazil; 3 Brazilian Biosciences National Laboratory, Brazilian Center for Research in Energy and Materials, Campinas, SP Brazil; 4 Retina Center of Minnesota, Minneapolis, MN USA

**Keywords:** Syringe, Intravitreal injection, Silicone oil droplets

## Abstract

**Background:**

Intravitreal silicone oil droplets have been found in the vitreous. The aim of this study is to compare the rates of silicone oil released by different brands of commonly used syringes for intravitreal injection after agitation by flicking.

**Methods:**

Three models of two brands of syringes were analyzed for their rates of silicone oil release: Saldanha Rodrigues (SR) 1 mL insulin syringe (SR, Brazil, syringe 1), Becton–Dickinson (BD) Plastipak 1 mL insulin syringe (Brazil, syringe 2), and BD Safety-Glide 1 mL insulin syringe (USA, syringe 3). All syringes were tested under four different conditions: positive control (fluid with addition of silicone oil) without agitation (group 1, n = 5); positive control with agitation (group 2, n = 3); fluid only without agitation (group 3, n = 5); and fluid only with agitation (group 4, n = 5). Masked graders performed all analyses using light microscopy.

**Results:**

All syringes (1, 2, and 3) released silicone oil droplets in the positive control group regardless of the agitation status (groups 1 and 2). When no oil was added and the syringes were not agitated, only syringe 1 released silicone oil droplets (40% of samples). After agitation, syringes 1 and 3 released silicone oil droplets in all samples. Quantitative analysis showed a significantly (*P *= 0.011; 11.2 ± 2.9 vs. 0.6 ± 0.9, respectively) higher mean number of silicone oil droplets released by syringe 1 after agitation compared to no agitation. Syringe 1 also had significantly (*P *= 0.002, 11.2 ± 2.9 vs. 0.0 ± 0.0 vs. 2.2 ± 0.8, respectively) more droplets than syringes 2 and 3 after agitation.

**Conclusions:**

Syringes commonly used for intravitreal injections frequently release silicone oil droplets when agitated by flicking, especially the SR insulin ones. We recommend that they not be agitated at the time of intravitreal injection and that the manufacturers consider producing syringes adapted for intraocular use.

## Background

Intravitreal injections are the most commonly performed intraocular treatment worldwide [1]. The number of indications for treatment has increased throughout the years when it became clear that intravitreal injections can slow, halt, or improve disease, leading to improved visual acuity. This treatment is important not only for vision but also for quality of life and work productivity.

Before 2005, intravitreal injections of antibiotics were administered off-label to treat infectious endophthalmitis, intravitreal injections of corticosteroids for inflammatory conditions and macular edema, and intravitreal injections of gas tamponades for pneumatic retinopexy [2]. Since anti-vascular endothelial growth factor (VEGF) agents were found to be effective to treat age-related macular degeneration (AMD), the number of intravitreal injections has skyrocketed [3].

Current on-label indications for intravitreal injections are AMD, macular edema secondary to diabetes and retinal vein occlusions, myopic choroidal neovascularization, and proliferative diabetic retinopathy [4, 5].

Many recent publications have reported that silicone oil droplets might be released by the syringe, leading to development of floaters [6–9]. Floaters can be so visually compromising to some individuals that vitrectomy has been performed to treat them. It is noteworthy that a more invasive surgery, although now safer, still is associated with an increased risk of complications, such as retinal tears and detachment, vitreous hemorrhage, and endophthalmitis [10]. Such vision-threatening diseases should not be acceptable as secondary to the presence of silicone oil droplets in the vitreous.

Some studies also have reported that some medications are more prone to cause ocular inflammation than others [11–13]. However, the causes are uncertain. Some reports have suggested the possible role of syringes used during intravitreal injections. Our group carried out a case–control study that associated inflammation after intravitreal injection of aflibercept (Eylea, Regeneron Pharmaceuticals, Tarrytown, NY) with the use of a specific brand of syringe [Saldanha Rodrigues (SR), Manaus, Brazil] [14]. In that series of patients, silicone oil droplets were observed in the vitreous of all six patients in whom inflammation developed. The authors speculated that there was a possible link between aflibercept and the inflammatory response to the silicone oil droplets.

These findings led us to perform another study to compare the rates of silicone oil release using different brands of commonly used syringes after agitation by flicking.

## Methods

Three models of two brands of syringes were analyzed for their rates of silicone oil release: syringe 1, SR 1-mL insulin syringe (lot #3719 K); syringe 2, BD Plastipak 1-mL insulin syringe [Becton, Dickinson (BD) and Co., Curitiba, Brazil, lot #6218341], and syringe 3, BD SafetyGlide Insulin 1-mL syringe (BD and Co., Holdrege, NE, lot #8010798).

The syringes were prefilled with distilled water at room temperature to 0.06 mL (backfilled either via their detachable needle or their own pre-attached needle). The air also was aspirated at 0.04 mL in order to facilitate injection of the entire amount of fluid, preventing retention in the dead space of the syringe.

All syringes were tested under four different conditions: group 1 (n = 5) positive control with addition of silicone oil without agitation; group 2 (n = 3), positive control with agitation; group 3 (n = 5), fluid only without agitation; and group 4 (n = 5), fluid only with agitation.

### Syringe preparation

After drawing distilled water and air, the syringe initially was kept upright in all groups with the needle side facing upwards. In groups 2 and 4, five consecutive flicks were performed. Groups 1 and 3 were not agitated. Subsequently, the syringe was turned 180° so that the needle faced down. In groups 2 and 4, the syringes were flicked 10 consecutive times to displace all fluid downward and the air upward. In groups 1 and 3, the syringes were moved gently to achieve the same air/fluid displacement. One investigator (G.B.M.) performed the previous steps in all syringes to avoid inter-examiner variability.

### Microscopic examination of syringe fluid

Ten drops of fluid were ejected continuously from each syringe onto glass slides (Yancheng Huida Medical Instruments Co., Jiangsu, China). The slides were viewed using a light microscope (Micronal S/A, São Paulo, Brazil). The slides were examined for oil droplets and photographed at 100X optical magnification (iPhone 6S mounted onto the microscope ocular) and an additional 50% digital magnification. Between three and five images of different drop per syringe were obtained at the same magnification.

**Fig. 1:**
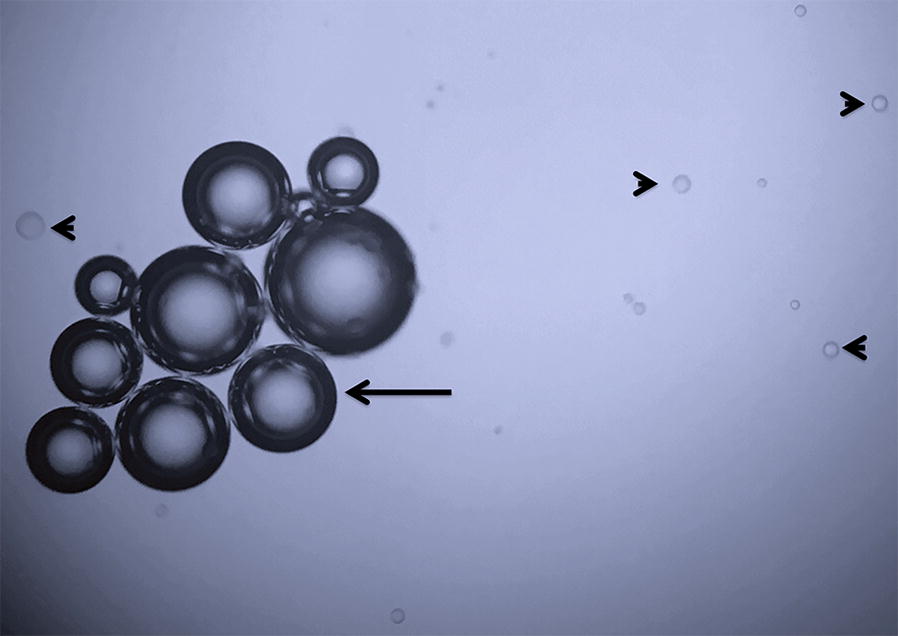
Silicone oil droplets (arrowhead) and air bubbles (arrow) in an agitated positive control (group 2) of syringe 3 (Becton–Dickinson)

The syringes were challenged with 1-mm droplets of 1000-cS silicone oil added to the tip of the syringe plunger to serve as positive controls. Additionally, the graders were instructed on how to identify silicone and air. The former usually appears as a round and transparent form with a thin border; the latter, which is commonly seen as a cluster, usually appears as a round form with a double grey ring that is distinguished easily from the former (Fig. 1).

Three masked graders evaluated the samples microscopically for the presence or absence of silicone oil and air, based on the criteria described previously. After the 3 graders reviewed the discrepancies together and came to a consensus, the data from the grader who was judged by the authors to be more accurate was chosen for analysis for the final comparison among the groups. Whenever silicone oil was identified, the numbers of drops were counted on each image. If any image from the syringe had silicone oil droplets, regardless of the number, it was considered as a positive result for that syringe.

### Statistical analysis

Statistical analyses were performed using SPSS 20.0 (IBM Corp, Version 20.0. Armonk, NY) and STATA 12 (StataCorp2011, College Station, TX). The data are expressed as the percentages of detection of silicone oil or air droplets per syringe. The mean numbers of silicone oil droplets and standard deviations also are shown. For quantitative analysis, only the images that presented the highest number of droplets were used for each syringe. The Kappa coefficient for categorical results and intra-class correlation for quantitative analysis were used to assess inter-examiner reproducibility. Fisher’s exact test compared nominal variables, such as the presence of oil, among the three brands of syringes. The mean number of silicone oil droplets among the groups was assessed using the Kruskal–Wallis’ non-parametric test. Dunn–Bonferroni’s post hoc test performed on each pair of groups. *P* = 0.05 was considered significant.

## Results

**Fig. 2:**
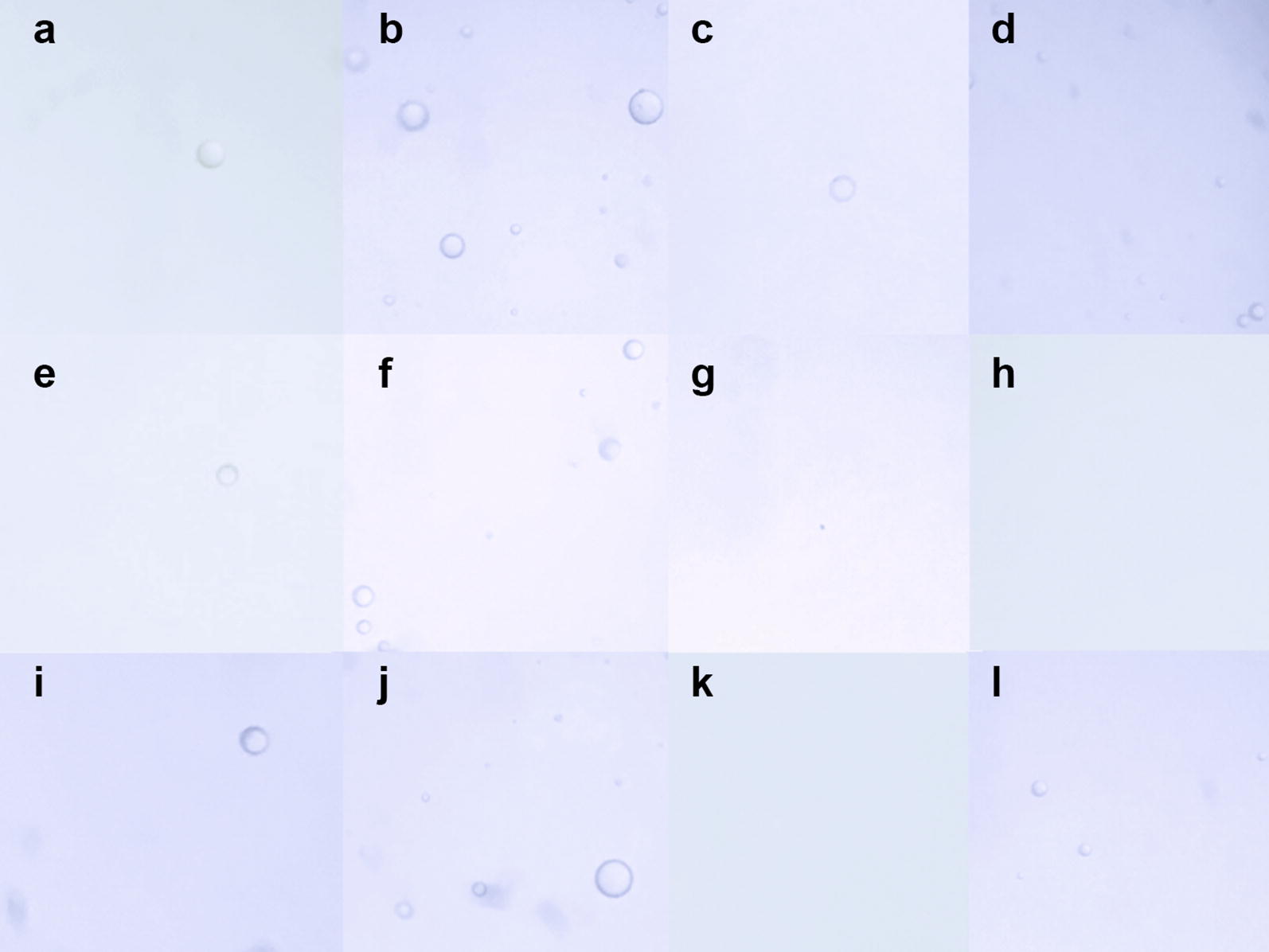
Images from syringes 1 [Saldanha Rodrigues (SR) 1-mL insulin syringe, **a**–**d**], 2 [Becton–Dickinson (BD) Plastipak 1-mL insulin syringe, **e**–**h**], and 3 [BD Safetyglide insulin syringe, **i**–**l**]. In syringe 1, oil droplets are seen in all groups: **a** Isolated silicone oil droplet in a positive control without agitation (group 1). **b** Multiple silicone oil droplets in a positive control with agitation (group 2). **c** Isolated silicone oil droplet in fluid-only without agitation (group 3). **d** Multiple silicone oil droplets in fluid-only with agitation (group 4). In syringe 2, no oil is seen in groups 3 and 4: **e** Positive control without agitation (group 1). **f** A positive control with agitation (group 2). **g** Fluid-only without agitation (group 3). **h** Fluid-only with agitation (group 4). In syringe 3, silicone oil is seen in three groups. **i** Isolated silicone oil droplet in a positive control without agitation (group 1). **j** Multiple silicone oil droplets in a positive control with agitation (group 2). **k** No silicone oil droplet in fluid-only without agitation (group 3). **l** Four silicone oil droplets in fluid-only with agitation (group 4)

Three graders obtained and assessed 206 images from 54 syringes. Illustrative images of each group are disclosed in Fig. 2. A high kappa coefficient (0.910 for the presence of oil and 0.819 for air) and intra-class correlation (graders 1 × 2, 0.915; graders 1 × 3, 0.77; graders 2 × 3, 0.736) were obtained for qualitative and quantitative analyses among the three masked graders. After challenging the divergent data among the graders, the three graders agreed that grader 2 was more likely to be correct. Therefore, the final data used in this study was derived from the measurements of grader 2.

Silicone oil droplets were observed in all syringes in the positive control groups (syringes 1, 2, and 3 in groups 1 and 2) (Table 1). With agitation (group 2), more silicone oil droplets were observed.

**Table 1:** Percentage of oil and air present in each group, according to the type of syringe

	Group				P*	*P* ^†^
	1	2	3	4		G3 × G4
Presence of oil						
Syringe 1, n (%)	5/5 (100)	3/3 (100)	2/5 (40)	5/5 (100)	0.037	0.067
Syringe 2, n (%)	5/5 (100)	3/3 (100)	0/5 (0)	0/5 (0)	0.001	1.000
Syringe 3, n (%)	5/5 (100)	3/3 (100)	0/5 (0)	5/5 (100)	< 0.001	0.008
Presence of air						
Syringe 1, n (%)	0/5 (0)	0/3 (0)	1/5 (20)	0/5 (0)	1.000	1.000
Syringe 2, n (%)	0/5 (0)	0/3 (0)	0/5 (0)	2/5 (40)	0.216	0.444
Syringe 3, n (%)	1/5 (20)	2/3 (66.7)	0/5 (0)	0/5 (0)	0.093	1.000

*n* number of samples, *P* level of significance

*Fisher’s exact test significance level

^†^Fisher’s exact test significance level considering groups G3 and G4

Without agitation of the fluid-only syringes (group 3), silicone oil droplets were seen only in syringe 1 (SR) (40% of the samples). Syringes 2 (BD Plastipak) and 3 (BD SafetyGlide) had no noticeable oil. With agitation (group 4), 100% of the samples from syringes 1 and 3 (*P *= 0.067 and *P *= 0.008, respectively, compared to no agitation in group 3) had identifiable silicone oil droplets. No samples from syringe 2 (group 4) had silicone oil droplets (Table 1). Air bubbles were seen in one sample from syringe 1 (group 3), two samples from syringe 2 (group 4), and three samples from syringe 3 (groups 1 and 2). This distribution did not reach significance (Table 1).

Tables 2 and 3 show the mean numbers of silicone oil droplets. More silicone oil droplets were visible in group 2 compared to group 1 (positive controls) in all three syringe types and these data reached significance. Syringe 1 had significant increase in the number of droplets in group 4 compared to group 3 (*P *= 0.004; 0.6 ± 0.9 to 11.2 ± 2.9). No silicone oil droplets were seen in syringe 2. An increase from 0.0 ± 0.0 to 2.2 ± 0.8 droplets was seen in syringe 3. However, this difference was not significant.

**Table 2:** Number of silicone oil droplets in each group, according to the type of syringe

	Group				P*	*P* ^†^
	G1 (n = 5)	G2 (n = 3)	G3 (n = 5)	G4 (n = 5)		G3 × G4
Mean number of oil droplets						
Syringe 1	2.5 ± 1.7	13.3 ± 6.8	0.6 ± 0.9	11.2 ± 2.9	0.004	0.011
Syringe 2	2.2 ± 2.2	10.0 ± 2.0	0.0 ± 0.0	0.0 ± 0.0	0.002	1.000
Syringe 3	3.4 ± 2.3	7.3 ± 4.2	0.0 ± 0.0	2.2 ± 0.8	0.004	0.263

The data are expressed as the mean ± standard deviation (SD)

*n* number of samples, *P* level of significance

*Kruskal–Wallis test significance level

^†^Dunn–Bonferroni’s multiple comparison adjusted significance level considering groups G3 and G4

**Table 3:** Number of silicone oil droplets comparing the syringe type, according to groups 3 and 4 (steady-state and agitation, no oil added)

Number of oil droplets	Syringe			P*
	1 (n = 5)	2 (n = 5)	3 (n = 5)	
Group 3, mean ± SD	0.6 ± 0.9	0.0 ± 0.0	0.0 ± 0.0	0.117
Group 4, mean ± SD	11.2 ± 2.9	0.0 ± 0.0	2.2 ± 0.8	0.002

The data are expressed as the mean ± standard deviation (SD)

*n* number of samples, *P* level of significance

*Kruskal–Wallis test significance level

## Discussion

The current study showed that flicking the syringes that are commonly used for intravitreal injections leads to a relevant release of silicone oil droplets. We performed this study for three major reasons: (1) some studies have shown that silicone oil was observed in the vitreous of patients treated with intravitreal injections [6–8, 14]; (2) we observed that patients who received injections with certain brands of syringes tended to have more oil droplets especially after the syringes were agitated (unpublished data); and (3) we speculated that a large amount of silicone oil released by syringes used to administer aflibercept might be associated with development of inflammation already reported [11–14].

A high kappa coefficient and intra-class correlation of the three masked graders assured the reliability and reproducibility of the findings. Consideration was given to whether the results obtained from masked grader 2 should be presented compared with the average. However, both results were essentially the same. Consequently, the group decided in retrospect that grader 2 was more accurate.

Emerson [15] recently reported that some BD insulin syringes also released silicone oil droplets. The experiments were performed under steady-state conditions of manipulation of the syringes. It was speculated that the absence of dead space in that specific syringe model was responsible. Theoretically, the dead space serves as a trap for high-resistance components such as silicone oil droplets. For instance, if the silicone oil lubrication were similar in two syringe types, fewer oil droplets would be expected in the syringe in the syringe with more dead space. Schargus et al. [16]. conducted steady-state biophysical analyses that showed that original prefilled ranibizumab (Lucentis, Genentech, Inc., South San Francisco, CA) glass syringes, original vials with aflibercept, and repacked ready-to-use plastic syringes filled with bevacizumab (Avastin, Genentech, Inc.) from a compounding pharmacy are similar regarding particulate purity and silicone oil microdroplet counts.

Flicking the syringe to dissociate fluid from air is a common practice among retina specialists in their routines (personal communication). Some authors also adopted this technique without knowing the risks involved. We first suspected that this was problematic when a cluster of six cases of inflammation following intravitreal injection of aflibercept developed at the clinical setting of some of the authors. A new brand of syringe (SR) had been introduced, and every patient in the series had many suspected silicone oil droplets in the vitreous. Additionally, all syringes had been agitated. Thereafter, a case–control study was performed, which reinforced the assumption that the new syringe was responsible for the inflammation [14]. In the current study, we proved that the SR syringes are associated with release of silicone oil droplets in 40% of the sample under steady-state conditions and in 100% after agitation by flicking.

These findings have implications not only for eyes treated with anti-VEGF injections but also after triamcinolone injections isolated and/or in combination with anti-VEGF drugs. When the syringes are filled with triamcinolone, the supernatant is separated completely from the steroid molecules in a few minutes [17]. Therefore, maneuvers to mix the steroid are very common. The authors argued that oil particles may stimulate the immunologic system, leading to vitritis or even pseudoendophthalmitis. It also is important to know that the sterilization method may be involved in protein aggregation and particle formation in polymer-based syringes, which also might affect the immune response [18]. SR and BD Plastipak syringes are sterilized by ethylene oxide, while BD SafetyGlide syringes undergo gamma radiation. Residues from the former process may include ethylene oxide and any of the following derivatives: ethylene chlorohydrin, ethylene bromohydrin, ethylene glycol, and diethylene glycol. Since the 1970s, it is well known that these residues can cause ocular inflammation [19]. Since SR syringes released a great deal of oil, the droplets may serve as the vehicle that delivers toxic substances from the sterilization process into the eye.

Many studies have reported an important association between the silicone oil–water interfaces (siliconized syringe walls), air–water interfaces (air bubbles), and agitation stress (occurring during end-over-end rotation) as triggers leading to protein aggregation and particles formation [20–22]. The highest particle concentrations were found in agitated, siliconized syringes containing an air bubble [21]. The particles formed in this condition consisted of silicone oil droplets and aggregated protein.

Different studies have reported the presence of silicone oil droplets in the vitreous [6–8]. Besides the suspected risk of inflammation [14], the presence of floaters can be so annoying that vitrectomy is required. The American Society of Retina Specialist 2018 Preferences and Trends Membership Survey showed that 60.4% of US respondents have seen silicone oil in the vitreous, while 5.2% already have performed vitrectomy to remove the oil [23]. Moreover, 1.8% reported that patients have sought legal action because of the presence of the floaters.

One limitation of the current study was the lack of a dye to stain the oil droplets. In a pilot study, the authors attempted to use Red Oil O (solvent red 27) dissolved in formaldehyde and propylene glycol. However, although this solution is not oily, it is immiscible in water, forming droplets that are mistaken for silicone oil. Therefore, the authors decided to carry out this experiment without use of a dye. Surgical silicone oil acted as a positive control that facilitated the correct identification of silicone oil droplets and air bubbles. The high inter-grader correlation confirmed the reproducibility and reliability. Another study limitation was the possibility of the non-standardized size of fluid drops on the glass slides, even though they were ejected in a continuous fashion and by the same investigator. Even though the risk was not eliminated, statistical analysis of both qualitative and quantitative data combined allowed for a clear and conclusive interpretation of the results. Finally, readers should consider that the findings were specific for specified lots of syringes and cannot be extrapolated to other lots.

Syringes commonly used for intravitreal injections frequently release silicone oil droplets when agitated by flicking, especially the SR insulin ones. We recommend that they not be agitated at the time of intravitreal injection and that the manufacturers consider producing syringes adapted for intraocular use.
